# Characterizing adolescent and youth‐friendly HIV services: a cross‐sectional assessment across 16 global sites

**DOI:** 10.1002/jia2.26437

**Published:** 2025-04-03

**Authors:** Lonnie Embleton, Tavitiya Sudjaritruk, Daisy Maria Machado, Belinda Chihota, Françoise Musabyimana, Julie Jesson, Edith Apondi, Thanyawee Puthanakit, Marco Tulio Luque, Nicola Ellen van Dongen, Gad Murenzi, Madeleine Amorissani‐Folquet, Zachary Kwena, Nicole Perreras, Vanessa Rouzier, Rita Lyamuya, Kim Anderson, Batya Elul, Valériane Leroy, Leslie A. Enane, Roxanne Martin, Kathryn Lancaster, Angela M. Parcesepe, Rachel Vreeman, IeDEA

**Affiliations:** ^1^ Arnhold Institute for Global Health Department of Global Health and Health Systems Design Icahn School of Medicine at Mount Sinai New York New York USA; ^2^ Division of Infectious Diseases Department of Pediatrics Faculty of Medicine Chiang Mai University Chiang Mai Thailand; ^3^ Clinical and Molecular Epidemiology of Emerging and Re‐emerging Infectious Diseases Cluster Faculty of Medicine Chiang Mai University Chiang Mai Thailand; ^4^ Pediatric Infectious Diseases Division Department of Pediatrics Escola Paulista de Medicina‐Universidade Federal de São Paulo (UNIFESP) São Paulo Brazil; ^5^ Centre for Infectious Disease Research in Zambia Lusaka Zambia; ^6^ Einstein‐Rwanda Research and Capacity Building Progam, Research for Development and Rwanda Military Hospital Kigali Rwanda; ^7^ Centre for Epidemiology and Research in POPulation Health (CERPOP) Inserm, Toulouse III University Toulouse France; ^8^ Moi Teaching and Referral Hospital Eldoret Kenya; ^9^ Department of Pediatrics Faculty of Medicine Chulalongkorn University Bangkok Thailand; ^10^ Servicio de Infectología, Departamento de Pediatría Hospital Escuela; Servicio de Infectología, Instituto Hondureño de Seguridad Social Tegucigalpa Honduras; ^11^ Empilweni Services and Research Unit, Department of Paediatrics and Child Health Rahima Moosa Mother and Child Hospital, University of the Witwatersrand Johannesburg South Africa; ^12^ Pediatric Department Centre Hospitalier Universitaire de Cocody Abidjan Côte d'Ivoire; ^13^ Research, Care and Treatment Programme Centre for Microbiology Research, Kenya Medical Research Institute Nairobi Kenya; ^14^ Department of Health AIDS Research Group, Research Institute for Tropical Medicine Manila Philippines; ^15^ Haitian Group for the Study of Kaposi's Sarcoma and Opportunistic Infections (GHESKIO) Port‐au‐Prince Haiti; ^16^ Morogoro Regional Referral Hospital Morogoro Tanzania; ^17^ Centre for Infectious Disease Epidemiology and Research School of Public Health University of Cape Town Cape Town South Africa; ^18^ Mailman School of Public Health Columbia University New York New York USA; ^19^ The Ryan White Center for Pediatric Infectious Disease and Global Health Department of Pediatrics Indiana University School of Medicine Indianapolis Indiana USA; ^20^ Department of Implementation Science Wake Forest University, School of Medicine Winston‐Salem North Carolina USA; ^21^ University of North Carolina at Chapel Hill Chapel Hill North Carolina USA

**Keywords:** adolescent, adolescent‐friendly health services, care services, HIV, implementation, transition

## Abstract

**Introduction:**

Adolescent and youth‐friendly health services (AYFHS) have been promoted as a best practice for adolescents and young people living with HIV (AYLH). However, thorough descriptions of AYFHS for AYLH remain scarce. We sought to characterize adolescent‐friendly HIV services in a global paediatric research consortium.

**Methods:**

Cross‐sectional data were collected from 16 global sites in the Adolescent and Young Adult Network of IeDEA (AYANI) of the International epidemiology Databases to Evaluate AIDS consortium between August 2020 and October 2022 using a standardized site assessment tool that collected data on clinic, patient and provider characteristics, differentiated care, and transition to adult services processes. Descriptive analyses characterized the health services available across the participating sites, using frequencies and proportions for categorical variables and medians and interquartile range for continuous variables. Data were analysed using RStudio.

**Results:**

Overall, 13 of 16 sites (81%) reported having dedicated adolescent services, which most often consisted of dedicated clinic days (62%, *n* = 8/13), primarily offered on weekdays. Across all sites, nurses and counsellors delivered services to adolescents. Over half of all clinics (69%, *n* = 11/16) reported offering health education to adolescents to facilitate adolescent health literacy. Peer educators and navigators were involved in delivering services at 62% of sites, primarily in those with dedicated adolescent services (69%, *n* = 9/13). There was limited integration of sexual and reproductive health services into HIV clinics for adolescents. With 63% of clinics conducting pregnancy screening, 50% providing family planning methods and 38% providing cervical cancer screening. Under half of all HIV clinics screened for physical abuse or violence (44%, *n* = 7/16) and sexual abuse or rape (38%, *n* = 6/16). A low proportion of clinics screened for risk factors related to young key populations, including drug use (56%, *n* = 9/16), homelessness (38%, *n* = 6/16) young men having sex with men (31%, *n* = 5/16) and transactional sex (31%, *n* = 5/16). Mental health screening for concerns was variable.

**Conclusions:**

Findings suggest gaps in AYFHS for AYLH across the HIV clinics included in this analysis. There is a vital need to design health services for AYLH that are accessible, equitable, and effective and meet the global standards for delivering high‐quality healthcare to adolescents.

## 1 INTRODUCTION

Significant progress has been made to achieve a decline in HIV acquisitions among young people, but there remain gaps in preventing new acquisitions and in meeting the diverse needs of adolescent and young people living with HIV (AYLH) [1, 2]. Globally, in 2019, there were approximately 1.7 million adolescents aged 10−19 years living with HIV, and young people aged 15−19 years accounted for two out of every seven new HIV acquisitions [1]. Adolescent and youth‐friendly health services (AYFHS) are a best practice to improve HIV viral suppression and optimize health outcomes for AYLH [2]. AYFHS aim to reduce or eliminate many of the barriers adolescents face when accessing HIV and other health services [2, 3]. AYFHS are those which are accessible, acceptable, equitable, appropriate and effective for all adolescents [4], and should align with World Health Organization's (WHO) eight global standards for quality healthcare services for adolescents, including (1) adolescent health literacy; (2) community support; (3) appropriate package of services; (4) providers’ competencies; (5) facility characteristics; (6) equity and non‐discrimination; (7) data and quality improvement; (8) adolescents’ participation [2, 5, 6]. The WHO has created HIV‐specific implementation considerations for AYLH, which outline that HIV service delivery approaches be integrated and align with WHO and national AYFHS standards, protocols and activities applicable to all adolescent health services [2, 6].

Adolescents face myriad burdens and barriers along the HIV prevention‐care continuum that impact their ability to access services, remain engaged in care and achieve viral suppression. These may include, but are not limited to, issues such as legal and policy barriers [1], gender inequities [7, 8], and stigma and discrimination outside of and within the health system [9, 10, 11]. For AYLH, health services must also be tailored to their specific needs related to HIV, including linkage to care, disclosure of their HIV status to others, retention in care, adherence and transition to adult services when they age out of paediatric or adolescent HIV care [2, 12, 13].

Health services for AYLH remain inadequate and evidence on the effectiveness, reach and coverage of AYFHS for AYLH is very limited [2, 3, 14–16]. Further, the quality and consistency of AYFHS and implementation of the WHO global standards may vary widely across service delivery sites [2]. While some exceptional examples of AYFHS for AYLH exist, such as Operation Triple Zero and Zvandiri [17, 18, 19], the WHO has stated there is still poor description and documentation in the literature of these models, and limited robust implementation and scale [2]. Detailed assessments are needed to understand what models of AYFHS for AYLH are currently implemented and to assess their quality and ability to meet global standards for AYFHS [2].

The availability of high‐quality integrated health services for AYLH is essential to reach the 95‐95‐95 targets [14]. HIV and other health services often remain separated for adolescents [2], and unresponsive health services remain a priority concern for AYLH, caregivers and healthcare providers [16]. It remains unclear how and if health services are being delivered to AYLH in an adolescent‐friendly manner, and if HIV clinics are adopting and implementing AYFHS that meet the WHO global standards. To address these gaps, we sought to describe and characterize how 16 clinics participating in the Adolescent and Young Adult Network of IeDEA (AYANI) are delivering health services to ALHV and implementing AYFHS standards.

## 2 METHODS

AYANI is a prospective cohort study nested within the International epidemiology Databases to Evaluate AIDS (IeDEA) to answer critical questions around HIV care outcomes; physical, mental and behavioural health; and the social circumstances of AYLH aged from 15 to 24 years. Launched in 2021, the AYANI study collects data on the characteristics, support, co‐morbidities and challenges that influence long‐term outcomes among consenting AYLH enrolled in HIV care at 16 clinics across six regions of IeDEA (Asia‐Pacific; Central and South America and the Caribbean [CCASAnet]; West Africa, Central Africa, East Africa and Southern Africa). Sites were selected for AYANI based on their caseload of AYLH and their interest in and capacity to participate in a prospective cohort study focused on AYLH. Sites include university hospitals, research institutes, referral, county and sub‐county hospitals, and health centres.

### 2.1 Study design and data collection

This cross‐sectional descriptive study relied on data from a standardized site survey distributed to 16 potential AYANI cohort sites between August 2020 and October 2022. The site survey drew upon items from prior IeDEA site assessments [20, 21, 22], with adaptations related to adolescent health and HIV care. The survey was designed to provide operational details needed to prepare and implement a common study protocol for AYANI and to describe the current state of AYLH care models, services available, management of transfer and transition to adult services, disclosure of HIV status to AYLH and other procedures related to adolescent HIV care. The site assessment survey is available upon request.

At each site, the survey was completed by a clinical provider or staff member knowledgeable about the site's HIV services and care for AYLH. Site surveys were completed electronically or on paper and then entered electronically using a REDCap database hosted at Indiana University. REDCap is a secure, web‐based software platform designed to support data capture [23, 24].

### 2.2 Statistical analysis

Descriptive analyses were conducted to characterize the health services available across the 16 HIV clinics delivering services to AYLH, using frequencies and proportions for categorical variables and medians and interquartile range for continuous variables. Analyses were stratified by sites that responded, “yes” versus “no” to the question “Does your HIV clinic offer dedicated services for adolescents?” to describe differences in service delivery across sites offering dedicated AYFHS and those that do not. To describe how sites are meeting a subset of the WHO global standards for delivering high‐quality healthcare services for adolescents [5], we utilized site survey data related to: adolescent health literacy (Standard 1); appropriate package of services (Standard 3); provider competencies (Standard 4); facility characteristics (Standard 5); and adolescents’ participation (Standard 8). Data were analysed using RStudio.

## 3 RESULTS

Site surveys were completed by 16 HIV clinics across 11 countries, including Brazil, Haiti, Honduras, Côte d'Ivoire, Kenya, Philippines, Rwanda, South Africa, Tanzania, Thailand and Zambia. Clinics were in urban (*n* = 14) or peri‐urban (*n* = 2) locations. The surveys were completed by service providers and facility staff, including doctor in‐charge (*n* = 5), doctor or medical officer working in clinic (*n* = 3), clinical officer or nurse in‐charge (*n* = 3), clinical officer or physician assistant (*n* = 2), programme officer in‐charge of health facility (*n* = 2) and a researcher coordinator (*n* = 1).

### 3.1 General characteristics of HIV clinics serving adolescents

Overall, 13 of 16 sites (81%) reported having dedicated adolescent services, and the majority (94%, *n* = 15) of sites reported having a working definition of adolescence (Table 1). Dedicated services for adolescents consisted of dedicated clinic days (62%, *n* = 8/13); dedicated clinic hours (38%, *n* = 5/13); dedicated providers (38%, *n* = 5/13); a dedicated room, department or venue (38%, *n* = 5/13); and school‐based services or community services (23%, *n* = 3/13). In facilities with dedicated space, this entailed a separate wing, hall or section at three sites, and a separate room in the HIV clinic at two sites. Dedicated services were most frequently provided on weekdays (69%, *n* = 9/13), followed by weekends (23%, *n* = 3/13). One site offered services during school breaks, and one had dedicated adolescent monthly support groups. For those without dedicated adolescent services (*n* = 3), one clinic reported that adolescents are treated in paediatric services until adulthood (>19 years), one site responded that adolescents are treated in adult services throughout adolescence (ages not specified) and one clinic treated all patients (i.e. paediatric, adolescent and adult) in the same venue, at the same times and by the same providers. Most commonly, across sites, nurses and counsellors delivered services to adolescents. Peer educators and navigators were involved in delivering services at 62% of sites, primarily in those with dedicated adolescent services (69%, *n* = 9/13).

**Table 1:** General characteristics of service delivery models for adolescents living with HIV across 16 sites with and without dedicated adolescent and youth‐friendly health services

		Has dedicated adolescent services	
General characteristics of health services for adolescents living with HIV	Total *N* = 16 *N* (%)	Yes *n* = 13 *n* (%)	No *n* = 3 *n* (%)
**HIV clinic has a working definition of adolescence**			
Yes	15 (94)	13 (100)	2 (67)
No	1 (6)	0 (0)	1 (33)
**Working definition median age range in years, (**min = 9 max = 29)	10−23	10−23	12.5−21.5
**Types of patients served at clinic**			
Children 0–9 years	16 (100)	13 (100)	3 (100)
Adolescents 10–19 years	16 (100)	13 (100)	3 (100)
Young adults 20–24 years	15 (69)	12 (92)	3 (100)
Adults 25+ years	11 (69)	8 (62)	3 (100)
**What cadre of providers deliver services to adolescents**			
Nurse	16 (100)	13 (100)	3 (100)
Counsellor/social worker	16 (100)	13 (100)	3 (100)
Peer educator/peer navigator	10 (62)	9 (69)	1 (33)
Community health worker	9 (56)	9 (69)	0 (0)
Paediatrician	9 (56)	8 (62)	1 (33)
Clinical officer or physician assistant	6 (38)	6 (46)	0 (0)
Non‐paediatrician doctor or medical officer	5 (31)	5 (38)	0 (0)
Psychologist	4 (25)	4 (31)	0 (0)
Other	2 (12)	2 (15)	0 (0)
**Clinical forms used during visits**			
Paediatric	10 (62)	7 (54)	3 (100)
Adolescent	5 (31)	5 (38)	0 (0)
Adult	4 (25)	2 (15)	2 (67)
Other	5 (31)	5 (38)	0 (0)

### 3.2 Retention, adherence, disclosure and transition to adult care practices

HIV clinics reported diverse care practices for adolescents for retention, adherence, disclosure and transition to adult care (Table 2). All HIV clinics reported using phone calls for following up with adolescents to support retention. Several (62%, *n* = 8/13) clinics with dedicated adolescent services reported using text messaging for follow‐up and a few used other digital approaches (social media and email) (15%, *n* = 2/13), whereas these strategies were not reported by clinics without dedicated adolescent services. Sites reported that phone messages, texts and WhatsApp groups were used to support adolescents navigating challenges with taking antiretroviral therapy (ART) (50%) and for appointment reminders (44%).

**Table 2:** HIV care practices for adolescents across 16 global sites

		Has dedicated adolescent services	
	Total *N* = 16 *N* (%)	Yes *n* = 13 *n* (%)	No *n* = 3 *n* (%)
**Mechanism in place for following up with adolescents living with HIV outside of the clinic**			
Phone calls	16 (100)	13 (100)	3 (100)
In‐person community visits	11 (69)	9 (69)	2 (67)
Dedicated staff	8 (50)	5 (38)	3 (100)
Text messaging	8 (50)	8 (62)	0 (0)
Other digital follow‐up (e.g. email, social media)	2 (12)	2 (15)	0 (0)
**How are phone messages, texts or WhatsApp groups used to support retention and/or adherence to ART**			
Follow‐up missed appointment	13 (81)	10 (77)	3 (100)
Support them to navigate challenges associated with taking ART	8 (50)	6 (46)	2 (67)
Reminders of upcoming appointments	7 (44)	7 (54)	0 (0)
Support them to navigate challenges associated with coming to the clinic	6 (38)	3 (23)	3 (100)
Reminders to take medicine	4 (25)	3 (23)	1 (33)
None of the above	2 (12)	2 (15)	0 (0)
**Disclosure counselling services for adolescents with perinatally acquired HIV**			
Yes	15 (94)	12 (92)	3 (100)
No	1 (6)	1 (8)	0 (0)
**Who provides HIV disclosure counselling services to adolescents with perinatally acquired HIV?**			
Counsellors/social workers	11 (69)	8 (62)	3 (100)
Paediatrician	10 (62)	9 (69)	1 (33)
Nurse	9 (56)	7 (54)	2 (67)
Clinical officers or physician assistant	5 (31)	4 (31)	1 (33)
Non‐paediatrician doctoral or medical officers	3 (19)	2 (15)	1 (33)
Peer educator/navigator	1 (6)	1 (8)	0 (0)
Community health worker	1 (6)	1 (8)	0 (0)
Psychologist	1 (6)	1 (8)	0 (0)
**Who participates in the disclosure process**			
Caregivers	15 (94)	12 (92)	3 (100)
Counsellors/social workers	12 (75)	9 (69)	3 (100)
Paediatricians, doctors, medical officers	10 (62)	9 (69)	1 (33)
Nurses	10 (62)	8 (62)	2 (67)
Family	8 (50)	5 (38)	3 (100)
Clinic officers or physician assistants	6 (38)	5 (38)	1 (33)
Peer educators/navigators	3 (19)	3 (23)	0 (0)
**Median age transition to adult services (Q3**−**Q1)**	22.5 (24.25−20.0)	21 (24.0−20.0)	25 (25.0−24.5)
**Processes used to support adolescents’ transition to adult HIV clinic**			
Assess adolescent readiness for transition	13 (81)	11 (85)	2 (67)
Individual counselling prior to transition	12 (75)	11 (85)	1 (33)
Disclose HIV status to adolescent	9 (56)	7 (54)	2 (67)
Identify providers who will be responsible for adolescent in adult HIV clinic	9 (56)	7 (54)	2 (67)
Communication between clinics before transition	8 (50)	7 (54)	1 (33)
Family counselling prior to transition	6 (38)	5 (38)	1 (33)
Individual counselling after transition	6 (38)	5 (38)	1 (33)
Communication between clinics after transition	6 (38)	5 (38)	1 (33)
Members of the paediatric adolescent HIV care team accompany the adolescent to initial appointment in adult HIV clinic	5 (31)	4 (31)	1 (33)
Draft individual transition plan	4 (25)	3 (23)	1 (33)
Family counselling after transition	3 (19)	2 (15)	1 (33)
Peer follow‐up	1 (6)	1 (8)	0 (0)
Do not know	1 (6)	1 (8)	0 (0)

Abbreviation: ART, antiretroviral therapy.

Almost all (94%, *n* = 15/16) sites provided disclosure counselling services for adolescents with perinatally acquired HIV, except for one clinic with dedicated adolescent services. Most commonly, counsellors and social workers (69%), paediatricians (62%) and/or nurses (56%) provide disclosure counselling services. Sites offering dedicated adolescent services reported a broader range of individuals providing disclosure counselling, including psychologists, community workers and peer educators or navigators, than those without dedicated services. Those participating in the disclosure process were parents or caregivers (94%), counsellors/social workers (75%), nurses (62%) and doctors (62%).

Across all clinics, they reported that patients move from paediatric or adolescent services to adult services at a median age of 22.5 years (Q3−Q1 = 24.25−20.0). The median age of transition was reported as 21 years (Q3−Q1 = 24.0−20.0) at sites with dedicated adolescent services and 25 years (Q3−Q1 = 25.0−24.5) at those without. Most sites reported that they assess an adolescent's readiness prior to transition to adult services (81%). Individual adolescent counselling before transition was reported by 11 (85%) sites providing dedicated services and one site (33%) without dedicated services. Less than half of the sites (38%, *n* = 6/16) provided individual counselling after transition; however, counselling services after transition may have been provided in the adult clinic. Few sites provided family counselling prior to (38%, *n* = 6/16) and after transition (19%, *n* = 3/16). One site reported peer follow‐up as a practice after transition to adult care. Just half of all clinics (50%, *n* = 8/16) reported communication between clinics before transition with fewer after transition (38%, *n* = 6/16). A quarter of sites reported drafting individual transition plans for AYLH.

### 3.3 Differentiated service delivery

Differentiated service delivery (DSD) models are patient‐centred models of care that simplify and tailor HIV services to better serve patients and reduce health system inefficiencies, and deliver ART in an alternative settings, such as the community [25]. DSD was reported by 12 of 16 sites (75%) (Table 3). DSD specifically for adolescents was reported by four sites (25%); three in those with dedicated adolescent services, and one without dedicated adolescent services. To be eligible for differentiated care, most sites reported that adolescents needed to be on ART (83%). Other eligibility requirements most frequently included being stable on ART (58%), adherent to ART (50%), being virally suppressed (50%), being aware of their HIV status (50%) and not being pregnant (50%). The most common DSD model reported was multi‐month prescribing (92%). Other DSD approaches were only available in HIV clinics with dedicated adolescent services, and included clinic‐based group care (60%, *n* = 6/10), community‐based group care (10%, *n* = 1/10) and community‐based individual care (10%, *n* = 1/10).

**Table 3:** Models of differentiated care delivery for adolescents living with HIV across 16 sites with and without dedicated adolescent and youth‐friendly health services

		Has dedicated adolescent services	
Characteristics of differentiated care delivery models for adolescents living with HIV	Total *N* = 16 *N* (%)	Yes *n* = 13 *n* (%)	No *n* = 3 *n* (%)
**Differentiated HIV care available for adolescents**			
Yes, adolescents are included in differentiated HIV care models for paediatrics	7 (44)	6 (46)	1 (33)
Yes, specifically for adolescents	4 (25)	3 (23)	1 (33)
Yes, adolescents are included in differentiated HIV care models for adults	1 (6)	1 (8)	0 (0)
No, but there are plans to implement	2 (12)	1 (8)	1 (33)
No, and there are no plans to implement	2 (12)	2 (15)	0 (0)
**Adolescent needs to be on ART to be eligible for differentiated care**	***N* = 12**	***n* = 10**	***n* = 2**
Yes	10 (83)	8 (80)	2 (100)
No	2 (17)	2 (20)	0 (0)
**Other eligibility requirements for differentiated care**			
Stable on ART	7 (58)	5 (50)	2 (100)
Adherent to ART	6 (50)	4 (40)	2 (100)
Virally suppressed	6 (50)	4 (40)	2 (100)
Aware of his/her HIV status	6 (50)	5 (50)	1 (50)
Not currently pregnant	6 (50)	4 (40)	2 (100)
Fully disclosed HIV to family	2 (17)	2 (20)	0 (0)
Over a certain age	1 (8)	1 (10)	0 (0)
Other	1 (8)	1 (1)	0 (0)
None of the above	3 (25)	3 (30)	0 (0)
**Type of differentiated care models**			
Multi‐month prescribing	11 (92)	9 (90)	2 (100)
Clinic‐based group care	6 (50)	6 (60)	0 (0)
Community‐based group care	1 (8)	1 (10)	0 (0)
Community‐based individual care	1 (8)	1 (10)	0 (0)

Abbreviation: ART, antiretroviral therapy.

### 3.4 AYFHS for AYLH

Implementation of global standards for AYFHS is presented in Table 4. Over half of all clinics (69%, *n* = 11/16) reported offering health education to adolescents to facilitate adolescent health literacy (Standard 1). All clinics without dedicated adolescent services report doing so, whereas 62% of those with dedicated adolescent services reported offering health education within the clinic. A few clinics (38%, *n* = 5/13) with dedicated adolescent services reported providing general health education via phone, texts or WhatsApp.

**Table 4:** WHO adolescent and youth‐friendly health service standards across clinics, with and without dedicated adolescent services across 16 HIV clinic sites

		Has dedicated adolescent services	
Domain	Total *N* = 16 *N* (%)	Yes *N* = 13 *n* (%)	No *N* = 3 *n* (%)
**Adolescents’ health literacy (Standard 1)**			
Offers education on health‐related topics (e.g. mental health, sexual health, HIV status disclosure)	11 (69)	8 (62)	3 (100)
Clinic uses phone messages, texts or WhatsApp groups to provide general health education	5 (31)	5 (38)	0 (0)
**Appropriate package of services (Standard 3)**			
**Clinic routine screening among adolescents**			
Disclosure of their HIV status to family/friends	16 (100)	13 (100)	3 (100)
Sexual activity	16 (100)	13 (100)	3 (100)
Disclosure of their HIV status to sexual partners	15 (94)	12 (92)	3 (100)
Unprotected sexual activity	15 (94)	12 (92)	3 (100)
Depression	14 (88)	11 (85)	3 (100)
Pregnancy	13 (81)	11 (85)	2 (67)
Need/desire for contraception	12 (75)	11 (85)	1 (33)
Dropping out of school	12 (75)	9 (69)	3 (100)
Alcohol use	12 (75)	10 (77)	2 (67)
Anxiety	11 (69)	8 (62)	3 (100)
Suicidal thoughts or behaviours	11 (69)	8 (62)	3 (100)
Loneliness/social isolation	9 (56)	7 (54)	2 (67)
Food insecurity	9 (56)	7 (54)	2 (67)
Drug use	9 (56)	9 (69)	0 (0)
Tobacco use	8 (50)	7 (54)	1 (33)
Homelessness	6 (38)	4 (31)	2 (67)
Physical abuse or violence	7 (44)	7 (54)	0 (0)
Sexual abuse or rape	6 (38)	6 (46)	0 (0)
Transactional sex	5 (31)	4 31)	1 (33)
Young men having sex with other men	5 (31)	4 (31)	1 (33)
Inhalant use	5 (31)	5 (38)	0 (0)
Other	1 (6)	1 (8)	0 (0)
**Sexual and reproductive health services routinely provided to adolescents in HIV clinic**			
STI screening	12 (75)	10 (77)	2 (67)
STI treatment	12 (75)	10 (77)	2 (67)
Sexual health counselling	12 (75)	10 (77)	2 (67)
Support for disclosure to partners	12 (75)	10 (77)	2 (67)
Pregnancy screening	10 (63)	8 (62)	2 (67)
Condom distribution	10 (63)	10 (77)	0 (0)
Family planning counselling	9 (56)	8 (62)	1 (33)
Provision, prescription or referral for oral contraceptive pills	8 (50)	6 (46)	2 (67)
Provision, prescription or referral for injectable contraceptives	8 (50)	6 (46)	2 (67)
Provision, prescription or referral for contraceptive implants	8 (50)	6 (46)	2 (67)
Cervical cancer screening	6 (38)	5 (38)	1 (33)
Provision, prescription or referral for IUD	5 (31)	3 (23)	2 (67)
HPV vaccination	4 (25)	3 (23)	1 (33)
**Clinic manages the HIV care of pregnant HIV‐positive adolescents**			
Yes, they are managed within our HIV clinic	5 (31)	3 (23)	2 (67)
No, they are managed in another HIV clinic within the health facility	4 (25)	4 (31)	0 (0)
No, they are referred to ANC within the health facility	4 (25)	3 (23)	1 (33)
No, they are referred to another health facility	2 (12)	2 (15)	0 (0)
Other	1 (6)	1 (8)	0 (0)
**Clinic manages antenatal care of pregnant HIV‐positive adolescents**			
Yes, they are managed within our clinic	5 (31)	3 (23)	2 (67)
No, they are referred to the antenatal care clinic within the health facility	10 (63)	9 (69)	1 (33)
No, they are referred to another health facility	1 (6)	1 (8)	0 (0)
**Provider competencies (Standard 4)**			
Have staff in your clinic received training for counselling related to disclosure of HIV status to adolescents with perinatally acquired HIV?			
Yes	15 (94)	12 (92)	3 (100)
No	1 (6)	1 (8)	0 (0)
**Health facility is “*friendly”* to adolescents (Standard 5)**			
Financial incentives (e.g. travel support, cash or credit for adherence or clinic attendance, access to lending programmes)	7 (44)	6 (46)	1 (33)
Support for education or remaining in school	7 (44)	5 (38)	2 (67)
Training on topics that are not related to health or vocation (e.g. life skills, personal finances)	6 (38)	5 (38)	1 (33)
Food or snacks	5 (31)	4 (38)	1 (33)
Inviting environment (e.g. music, TV, artwork)	4 (2)	4 (38)	0 (0)
Vocational training	3 (19)	3 (23)	0 (0)
Drama/theatre, sporting or art activities	3 (19)	2 (15)	1 (33)
Other	3 (19)	3 (23)	0 (0)
None of the above	1 (6)	1 (8)	0 (0)
**Adolescents’ participation (Standard 8)**			
Facility has adolescent peer educators/navigators	13 (81)	11 (85)	2 (67)
Peers deliver services to adolescents in the clinic	10 (62)	9 (69)	1 (33)
Peers coordinate clinic‐based group care	3 (19)	3 (23)	0 (0)
Peers provide disclosure counselling	1 (6)	1 (8)	0 (0)
Peers participate in disclosure process	3 (19)	3 (23)	0 (0)

Abbreviations: ANC, antenatal care; IUD, intrauterine device; STI, sexually transmitted infection.

An appropriate package of services to adolescents includes information, counselling, diagnostic, treatment and care services that fulfils the needs of all adolescents (Standard 3). HIV clinics screened adolescents for a wide range of health and socio‐economic issues that may impact their care (Table 4). Across sites, all clinics screened for disclosure of HIV status to friends and family and sexual activity among adolescents. Under half of all HIV clinics screened for physical abuse or violence (44%, *n* = 7/16) and sexual abuse or rape (38%, *n* = 6/16), and only among those reporting dedicated adolescent services. Likewise, a low proportion of clinics screened for issues related to young key populations, including drug use (56%, *n* = 9/16), homelessness (38%, *n* = 6/16), young men having sex with men (31%, *n* = 5/16) and transactional sex (31%, *n* = 5/16).

Sexual and reproductive health (SRH) services integrated into HIV clinics for adolescents were varied. Most clinics reported offering sexual health counselling (75%, *n* = 12/16), sexually transmitted infection (STI) screening (75%, *n* = 12/16) and STI treatment (75%, *n* = 12/16). Just over half of all clinics reported the provision of family planning counselling (56%, *n* = 9/16), with the majority being clinics with dedicated adolescent services (62%, *n* = 8/13). Half of all sites (50%, *n* = 8/16) reported the provision of oral, injectable and implant contraceptive regimens, and few reported providing intrauterine devices (31%, *n* = 5/16). A small proportion of clinics reported offering cervical cancer screening (38%, *n* = 6/16) and a quarter human papillomavirus (HPV) vaccination (25%, *n* = 4/16). Most clinics (69%, *n* = 11/16) reported that they do not manage HIV or antenatal care for pregnant adolescents.

Almost all clinics (94%, *n* = 15/16) reported that providers received training for disclosure of HIV status to adolescents with perinatally acquired HIV (Standard 4). Across sites, there was limited availability of other adolescent‐friendly support and activities to facilitate a welcoming and acceptable environment for adolescents (Standard 5). Most commonly, clinics reported the availability of financial incentives (44%, *n* = 7/16), support for education (44%, *n* = 7/16), life skills (38%, *n* = 6/16) and food or snacks (31%, *n* = 5/16). Lastly, most clinics (81%, *n* = 13/16) reported having adolescent peer educators/navigators (Standard 8). However, adolescent involvement in aspects of service provision was largely limited to clinics that reported having dedicated adolescent services.

## 4 DISCUSSION

Our analysis demonstrated varying implementation of robust AYFHS for AYLH across the 16 global sites delivering HIV care. This analysis identified implementation gaps in meeting the existing WHO global standards for delivering quality healthcare to adolescents, regardless of reporting offering dedicated adolescent services. While 13 of 16 HIV clinics reported that they offered dedicated adolescent services, services were highly varied and were limited in their “adolescent‐friendliness.” Further, across all sites, approaches to support adolescents’ transition to adult care were limited.

Despite comprehensive guidelines and tools for the design, implementation and evaluation of AYFHS from the WHO [2, 4, 5, 26, 27, 28], this analysis identified key implementation gaps in global standards for delivering AYFHS for AYLH, notably in the delivery of an appropriate package of services. Similar to findings previously reported in the broader paediatric IeDEA cohort [22], there are critical gaps in access to broader services for adolescents, particularly with respect to routine screening and SRH. Importantly, this analysis demonstrated minimal routine screening for important issues that impact young key populations, such as harmful substance use, same‐sex partnerships, transactional sex and housing instability, all of which impact HIV treatment outcomes [29, 30]. The WHO has designed guidelines to meet the needs of key populations in acceptable and effective health services [29]. Screening for issues impacting young key populations, who are disproportionately affected by HIV, should be routinely integrated into care for all adolescents, with consideration for the local social, cultural and legal context. Further, under half of all clinics reported screening for physical or sexual violence, despite violence being a substantial risk factor for HIV acquisition and a major barrier to adherence to ART [31].

Integration of SRH services for adolescents in the HIV clinics included in this site assessment was inadequate. Yet, adolescence is generally the period of development when young people sexually mature, and may become sexually active [32]. AYLH express desires to engage in romantic relationships and sexual activities [16, 33–35], and several studies report AYLH engaging in sexual activity [36, 37]. SRH services should be available for AYLH to promote sexual wellbeing and care throughout and after pregnancy [38]. For AYLH, the integration of SRH services into their HIV care is important for destigmatizing sexuality and creating a healthy foundation for sexual wellbeing [39], as well as to reduce the risk of onward horizontal and vertical transmission. AYLH have substantial unmet sexual healthcare needs [40], and have requested for SRH services [16]. Rates of other key preventive health promotion strategies, such as HPV vaccination, also remain low [41]. Offering integrated SRH services within HIV care for adolescents may reduce high‐risk sexual practices (e.g. unprotected sex) and unintended pregnancies for AYLH [36, 42].

Clinics with dedicated adolescent services primarily reported that this consisted of offering dedicated clinic days, and that dedicated services were most frequently offered on weekdays. The provision of services primarily on weekdays has been reported as a structural barrier to care for AYLH, as it conflicts with school attendance and may force AYLH to choose between their health and academics, thereby impacting retention and school performance [43], and potentially contributing to stigma [44]. Convenient clinic operating hours enable accessibility and are a core component of WHO Standard 5, health facility characteristics [2], and flexible opening hours outside of regular clinic hours, such as evenings after school or weekends may facilitate retention in care [2, 43, 45, 46]. Similarly, only a small number of clinics reported that they have dedicated space or providers to support AYLH. Yet, offering a safe space for HIV care and psychosocial discussions are important facility characteristics for the provision of AYFHS [2]. In addition, less than half of the HIV clinics with dedicated services reported offering specialized features to make HIV care more “adolescent‐friendly,” such as support for education, an inviting environment, or other recreational or creative activities. Taken together, these findings suggest that even among HIV clinics that report offering dedicated adolescent services, several gaps exist in the implementation of comprehensive AYFHS for AYLH [2, 3].

Transition from paediatric to adult care for AYLH is an important challenge, which can have a significant impact on retention in care and other health outcomes [12, 47]. Transition of care for AYLH may occur between the ages of 18−25 years [48], and involve changes in clinic location, providers and/or models of care, and a shift whereby adolescents take on responsibility to manage their own HIV care [12]. If the transition process is fragmented or the AYLH is not well‐supported, gaps in care may result in non‐adherence to ART, viral resistance and loss to follow‐up for AYLH [47]. Programmes and policies have been developed and tested to support AYLH to transition from paediatric to adult care [48, 49]. Several components of a successful transition process have been proposed including supporting caregiver readiness, communication between clinics, adolescent readiness assessment, peer support, flexibility to return to adolescent or paediatric clinics, group transition, and holistic clinical and psychosocial support and counselling throughout the process [12, 48].

Assessing readiness for transition should include ensuring the adolescent has sufficient HIV literacy, established capacities for self‐management and communication of their care needs, and care support. Adolescent readiness also involves ensuring the adolescent is on an optimized ART regimen and stable on treatment. Finally, a readiness assessment should consider the adolescents’ willingness to transition and their school, social, and family‐related concerns [48]. The present analysis identified that assessing adolescent readiness was reported among most HIV clinics as a component of the process to support transition to adult care. However, gaps were identified for supporting a successful transition process. A limited number of HIV clinics reported using communication between clinics before and after transition. Few sites reported using family counselling. While three‐quarters of clinics reported providing individual counselling before transition, under half reported individual counselling after transition, and only a quarter of clinics reported drafting individual transition plans. Given that transition from paediatric or adolescent to adult care is a critical period for AYLH and requires a planned and ongoing process involving a multidisciplinary team, including peers and caregivers to provide support to a successful transition, our findings suggest key gaps in supporting AYLH to transition to adult care across the 16 global sites included in this analysis.

While this analysis richly described the services available across 16 global sites for AYLH, it has several limitations. First, the site assessment was not explicitly designed to assess the WHO global standards for AYFHS for AYLH [2], and additional research is needed to thoroughly examine how HIV clinics are delivering AYFHS according to WHO standards, which are currently being revised. Next, while global in reach, representing 11 countries spanning Africa, South and Central America, and South‐East Asia, our assessment was limited to self‐reported data from 16 well‐resourced HIV clinics, and therefore, our findings may not be representative of models of HIV care for adolescents within these or other countries and contexts. Notwithstanding these limitations, this analysis also has strengths. The geographic scope of the analysis fills a significant gap in the literature to provide a detailed description of what AYFHS look like for AYLH in well‐resourced sites globally.

## 5 CONCLUSIONS

Overall, these findings suggest implementation gaps in adolescent health services for AYLH across the global sites included in this analysis. There is a vital need to augment efforts to ensure health services for AYLH are meeting global standards for delivering high‐quality healthcare to adolescents. Implementation gaps in delivering high‐quality AYFHS for living with HIV impede efforts to reach global targets for ending the HIV epidemic.

## COMPETING INTERESTS

The authors have no conflicts of interest to declare.

## AUTHORS’ CONTRIBUTIONS

LE conceptualized and executed the analysis and led the drafting of the initial manuscript. RV led the conceptualization of the study design, supervised study contact across all regions, and participated fully in developing and revising the manuscript. TS, DMM, BC, FM, JJ, EA, TP, MTL, NEvD, GM, MA‐F, ZK, NP, VR, RL, KA, BE, VL, LAE, KL and AMP participated in the study design, survey construction and implementation; and contributed to, reviewed, and approved the final manuscript. RM provided support for study design, survey construction, data collection and management, regional approvals, and contributed to, reviewed, and approved the final manuscript.

## FUNDING

The International epidemiology Databases to Evaluate AIDS (IeDEA) Consortium is supported by the U.S. National Institutes of Health's National Institute of Allergy and Infectious Diseases, the Eunice Kennedy Shriver National Institute of Child Health and Human Development, the National Cancer Institute, the National Institute of Mental Health, the National Institute on Drug Abuse, the National Heart, Lung, and Blood Institute, the National Institute on Alcohol Abuse and Alcoholism, the National Institute of Diabetes and Digestive and Kidney Diseases, and the Fogarty International Center. AYANI‐participating regions: Asia‐Pacific, U01AI069907; CCASAnet, U01AI069923; Central Africa, U01AI096299; East Africa, U01AI069911; Southern Africa, U01AI069924; West Africa, U01AI069919. Informatics resources are supported by the Harmonist project, R24AI24872. This work is solely the responsibility of the authors and does not necessarily represent the official views of any of the institutions mentioned above.

## DISCLAIMER

This work is solely the responsibility of the authors and does not necessarily represent the official views of any of the institutions mentioned above.

## Data Availability

Please visit www.iedea.org for additional information about collaborating with the IeDEA global consortium.
